# Recruitment, data collection, participation rate, and representativeness of the international cross-sectional PRICOV-19 study across 38 countries

**DOI:** 10.1186/s12875-024-02438-w

**Published:** 2024-06-27

**Authors:** Athina Tatsioni, Peter Groenewegen, Esther Van Poel, Kyriaki Vafeidou, Radost Assenova, Kathryn Hoffmann, Emmily Schaubroeck, Stefanie Stark, Victoria Tkachenko, Sara Willems

**Affiliations:** 1 Research Unit for General Medicine and Primary Health Care, Faculty of Medicine, School of Health Sciences, University of Ioannina, 45110 Ioannina, Greece; 2 Netherlands Institute for Health Services Research (Nivel), 3500 BN Utrecht, The Netherlands; 3 Department of Sociology, Utrecht University, 3584 CS Utrecht, The Netherlands; 4 Department of Human Geography, Utrecht University, 3584 CS Utrecht, The Netherlands; 5 Department of Public Health and Primary Care, Ghent University, 9000 Ghent, Belgium; 6 Department of Urology and General Practice, Faculty of Medicine, Medical University of Plovdiv, Plovdiv, Bulgaria; 7 Unit Health Services Research and Telemedicine in Primary Care, Medical University of Vienna, Vienna, Austria; 8 Institute of General Practice, Friedrich-Alexander University Erlangen-Nürnberg (FAU), 91054 Erlangen-Nuremberg, Germany; 9 Department of Family Medicine, Shupyk National Healthcare University of Ukraine, Kiev, Ukraine

**Keywords:** COVID-19, Family medicine, PRICOV-19 study, Primary health care, Quality of care, Recruitment, Representativeness

## Abstract

**Background:**

Recruitment for surveys has been a great challenge, especially in general practice.

**Methods:**

Here, we reported recruitment strategies, data collection, participation rates (PR) and representativeness of the PRICOV-19 study, an international comparative, cross-sectional, online survey among general practices (GP practices) in 37 European countries and Israel.

**Results:**

Nine (24%) countries reported a published invitation; 19 (50%) had direct contact with all GPs/GP practices; 19 (50%) contacted a sample of GPs /GP practices; and 7 (18%) used another invitation strategy. The median participation rate was 22% (IQR = 10%, 28%). Multiple invitation strategies (*P*-value 0.93) and multiple strategies to increase PR (*P*-value 0.64) were not correlated with the PR. GP practices in (semi-) rural areas, GP practices serving more than 10,000 patients, and group practices were over-represented (*P*-value < 0.001). There was no significant correlation between the PR and strength of the primary care (PC) system [Spearman’s r 0.13, 95% CI (-0.24, 0.46); *P*-value 0.49]; the COVID-19 morbidity [Spearman’s r 0.19, 95% CI (-0.14, 0.49); *P*-value 0.24], or COVID-19 mortality [Spearman’s r 0.19, 95% CI (-0.02, 0.58); *P*-value 0.06] during the three months before country-specific study commencement.

**Conclusion:**

Our main contribution here was to describe the survey recruitment and representativeness of PRICOV-19, an important and novel study.

**Supplementary Information:**

The online version contains supplementary material available at 10.1186/s12875-024-02438-w.

## Introduction

Online surveys, already very common in (international) opinion research and organisational research [1], became a crucial tool during the COVID-19 pandemic as an alternative to traditional postal surveys, allowing for the collection of real-time data despite the global restrictions that were put in place [2]. Online surveys come with various advantages: ease of use for the respondent, ease of data entry for the researcher, low cost, a wide range of options for disseminating the survey, and flexibility of question design [3]. However, recruitment for surveys, not only virtual, to ensure an adequate response rate and a representative sample for the target population has always been a great challenge [4]. Low recruitment rates are common and can impact data quality, resulting in representativeness problems comparable to convenience samples [5–8].

In this paper, we report on the recruitment strategies, data collection, participation rates and representativeness of the PRICOV-19 study, an international comparative, cross-sectional, online survey among general practitioners (GPs) in 37 European countries and Israel [9]. The PRICOV-19 study collected information using an online self-reported questionnaire on how GP practices were organised during the COVID-19 pandemic to guarantee safe, effective, efficient, patient-centred, and equitable care; and assessed the shift in roles and tasks in practice and the wellbeing of staff members during the pandemic. The data collection took place between November 2020 and June 2021 for most participating countries. Due to its scale and multi-country design, the PRICOV-19 study also explored which practice and health care system characteristics were associated with better care and how GP practices coped with COVID-19 related challenges. Its results may suggest which features of health systems and general practice organization must be reinforced to prepare primary care systems across Europe against future pandemics.

The main contribution of this paper was to describe the survey recruitment and representativeness of the PRICOV-19 study, an important and novel study, and reflect on what could be done in the recruitment, and data collection for future large-scale cross-country studies in primary care. Specifically, this paper has five aims:

1. to report the strategies used to recruit GP practices within the PRICOV-19 study;
2. to report the resulting participation rate per country and whether participating countries reached the target number of GP practices requested in the PRICOV-19 protocol;
3. to explore associations between recruitment strategies and participation rates;
4. to assess the representativeness of the actual response group referring to whether the GP practices in the response group represents the population GP practices regarding background characteristics of the population of GP practices in the country.
5. to explore whether participation rates among countries were correlated to country health system characteristics, i.e., to the strength of the primary care (PC) system [10], the burden due to the COVID-19 pandemic during the first wave (urgency effect), and the burden in the months before the survey (workload effect).

The added value of our analysis is threefold. First, it provides important information for readers of the separate papers based on the data of the PRICOV-19 study, some of which have been published already [11–21], others in preparation. Specifically, our work provides adequate information on recruitment strategies, participation rates, and representativeness that may facilitate the interpretation of the PRICOV-19 findings after considering potential biases and generalisability. Secondly, the experiences of the PRICOV-19 study are relevant to the design of future international comparative surveys of general practice, such as the PaRIS survey developed by the OECD [22]. Finally, exploring whether the participation rates are correlated to country health system characteristics may provide insights about potential factors that can be considered in the design of future multi-country survey studies in PC.

## Methods

### Ethical approval

The study was conducted according to the guidelines of the Declaration of Helsinki. The Research Ethics Committee of Ghent University Hospital approved the protocol of the PRICOV-19 study (BC-07617). Research ethics committees in the different partner countries gave additional approval if needed in that country. All participants gave informed consent on the first page of the online questionnaire. All data were anonymised, and all raw data that could lead to the identification of the respondents were permanently removed.

### PRICOV-19 study design

The PRICOV-19 study was initiated by Quality and Safety Ghent, the expert centre on quality and patient safety in PC and transmural care at Ghent University (Belgium). For this study, an international research consortium with over 45 universities and research institutes from 38 countries was formed. The study was conducted in 37 European countries: Austria, Belgium, Bosnia and Herzegovina, Bulgaria, Croatia, Cyprus, Czech Republic, Denmark, Estonia, Finland, France, Germany, Greece, Hungary, Iceland, Ireland, Italy, Kosovo*, Latvia, Lithuania, Luxembourg, Malta, Moldova, The Netherlands, North Macedonia, Norway, Poland, Portugal, Romania, Serbia, Slovenia, Spain, Sweden, Switzerland, Turkey, Ukraine, and the United Kingdom; and in Israel.

A self-administered, web-based questionnaire was used to collect information on GP practices. The average time of the questionnaire completion was 20 min. The intention was that only one person per practice (usually a GP or a practice manager) would respond to avoid duplication of practice characteristics. A detailed description of the development and validation of the questionnaire is in the study protocol, including a pilot study in Flanders (Belgium) [9]. The final version of the questionnaire consisted of 53 items divided into six topics: (a) infection prevention; (b) patient flow for COVID and non-COVID care; (c) dealing with new knowledge and protocols; (d) communication with patients; (e) collaboration; (f) wellbeing of the respondent; (g) and characteristics of the respondent and the practice [9]. The Research Electronic Data Capture (REDCap) platform [23] was used for data collection. Specifically, we used this software to host the questionnaire in all languages, send out invitations to the national samples of GP practices, and securely store the participants’ answers [23].

### Additional data collection on the sample selection and recruitment approaches

In each country, the consortium partner(s) were requested to recruit GP practices following two considerations: (a) a recruitment procedure based on drawing a random sample among all GP practices in the country was preferred over convenience sampling and (b) each unit of analysis should represent a practice (one response per practice was required). PRICOV-19 aimed to sample between 80 and 200 GP practices per country, depending on the size of the country [9]. Since the PRICOV-19 study was not externally funded, consortium partner(s) in each country worked voluntarily.

Partners logged all the steps in the recruitment procedure, including strategies to increase the participation rate in an online structured questionnaire (see Additional file 1 Table S1). In addition, all partners were encouraged to report any additional information they considered relevant to the invitation strategy and the strategy they used to increase the participation rate.

### Extracted variables

Based on the database, including the data on recruitment strategies as reported by country coordinators, we extracted the invitation strategy category, i.e., published invitation and where it was published (i.e., newsletter, social media, Medical Association website, and multiple venues), direct contact with all GP practices in the country, contact with a sample of GP practices, and other strategies. For countries that contacted all GP practices or invited a sample of GP practices, we also extracted the origin of the contact list (i.e., government or governmental organisation, National College of GPs, previous study, GPs who were known to or had collaborated with the country coordinator before, another origin). For countries that did not use a published invitation, we recorded how the invitation was sent (i.e., by e-mail, post, phone call, or other). For countries that contacted a sample of GP practices, we also recorded the sample selection process (random, convenience, or mixed selection process), whether stratification was used, and if so, stratified for what. For all invitation strategies, we extracted who was reached (GPs /GP trainees, practice managers, other) and whether all regions of the country were reached, and if they reached a part of the country, which regions were included. We also extracted the estimated number of GP practices invited to participate by each invitation strategy; we extracted the number of GP practices invited to participate separately for countries that used a single strategy and countries that used multiple strategies. Finally, we recorded whether participating countries used strategies to increase the participation rate, and if so, the strategy they used (reminders, financial incentives, keeping the participant informed about the study results, offering accreditation points to the participant, other). For countries that used reminders, we also recorded the number of reminders and how they were sent (by e-mail, by phone, other). Finally, we reported the number of participating countries that used a single strategy and countries that used multiple strategies to increase participation.

Based on the merged final database that included the survey data from all participating countries (response population), we extracted the variables on the GP practice location, GP practice size, and GP practice type. For each participating country, we calculated the proportion of GP practices per location category [urban vs. town/suburbs vs. (semi-) rural]; per category of the number of patients registered or – when there was no patient register – the size of population in the area served (up to 2500 vs 2501 to 10,000 vs. 10,001 to 50,000 vs more than 50,000); and per practice type [i.e., practice with one GP (solo) vs. practice with two GPs (duo) vs. group practice].

### Additional data

To assess representativeness of the response groups per country, we requested additional information (see Additional file 1 table S2) from each country coordinator on the total number of GP practices in the country, the proportion of GP practices per location [urban vs town/suburbs vs (semi-) rural], per practice size category (GP practices with up to 2,500 registered patients, with 2,501 to 5,000 patients, with 5,001 to 10,000 patients, with 10,001 to 50,000 patients, and GP practice with more than 50,000 registered patients), and per practice type [practice with one GP (solo), practice with two GPs (duo), and group practice]. Country coordinators provided this from official registries or – if there were no official population data – they gave an estimate.

In addition, for each country, we used the strength of the PC system based on published indicators [24–26] (see Additional file 1 table S3). Specifically, the focus of the set of indicators to assess the strength of the PC system was on the structure (governance, financing, workforce development), process (access, continuity, coordination, comprehensiveness) and outcome (quality, efficiency, equity) of PC systems [25]. The data were collected across Europe by reviewing (inter)national literature and statistical databases, and consulting panels of national experts [25]. For each country, we also recorded cumulative COVID-19 cases (per 1 million) and COVID-19 mortality (per 1 million) during the first wave (May 2020); and COVID-19 incidence (per 1 million) and COVID-19 mortality (per 1 million) during the three months before starting the data collection for the PRICOV-19 study [27] (see Additional file 1 table S3).

### Outcomes

First, we described the invitation strategies used to recruit GP practices across 38 countries. We categorised invitation strategies with a publicly advertised invitation, e.g., through a newsletter or a publicly available website, direct invitation of all GP practices, invitation of a sample of GP practices, and other invitation strategies.

Second, we calculated the participation rate as the ratio of the number of GP practices that at least filled in the first part of the questionnaire to the number of GP practices that received an invitation to participate in the study [28], as reported by the country coordinator. We also reported whether the number of participating GP practices in each country reached the requested aim in the PRICOV-19 protocol.

To explore representativeness, we examined how well the PRICOV-19 response group represents the population in terms of the distribution in the population of GP practices by location, practice size, and type of practice for each country, as reported by country coordinators.

Finally, we explored whether participation rates varied among countries based on the strength of the PC system and intensity of the COVID-19 pandemic as indicated by the COVID-19 morbidity and mortality during the first three months since the onset of the pandemic and during the three months before the start of PRICOV-19 data collection in each country.

### Statistical analyses

Data were presented as absolute numbers and percentages for binary and categorical variables, and as median with interquartile range (IQR) for continuous variables. We described the estimates of GP practices invited to participate per invitation strategy category. We separately described the estimates of GP practices reached in countries that used multiple invitation strategies. To explore whether the use of multiple invitation strategies and the use of multiple strategies to increase participation rate were correlated to the participation rate, we presented the number of countries using multiple invitation strategies and the number of countries using multiple strategies to increase the participation rate per participation rate quartile. We hypothesised that the use of multiple invitation strategies or multiple strategies to increase participation correlated with higher participation rates. Comparisons were performed using the Pearson Chi-Square test or the Fisher’s Exact test as appropriate.

To examine how well the PRICOV-19 response group represents the population in terms of the distribution of the population of GP practices according to location, practice size, and type for each country, as reported by country coordinators, we used the standard approach to conduct the one-sample chi-square test [29]. To have a pragmatic approach given the challenges in multi-country surveys among GPs, we assumed that a less than 10%-point difference is reasonably close to the population distribution. We further explored representativeness by comparing countries that reached the target number of participating GP practices; countries that invited only a random sample; countries that invited a mixed sample including a randomly selected sample; and countries that invited all GP practices.

To explore whether country health system characteristics were correlated to the participation rate, we compared the strength of the primary health care system, COVID-19 morbidity, and mortality during the first three months since the onset of the COVID-19 pandemic and during the three months before the country-specific study commencement and participation rate in each country. We hypothesised that a less strong primary care (PC) system [10], a higher burden due to the COVID-19 pandemic during the first wave (urgency effect), and a lower burden in the months before the survey (workload effect) might be correlated to a higher participation rate. Comparisons were performed using the Spearman rank correlation coefficient r with 95% confidence intervals (CIs).

For all comparisons, the null hypothesis was that there is no difference among countries per participation rate quartile, and we considered statistically significant a two-tailed *P*-value less than 0.05. All analyses were performed with IBM SPSS Statistics for Windows, Version 26.0. (IBM Corp, Armonk, NY, USA), and Microsoft Excel, MS Office 2019 (Microsoft Corp, Redmond, Washington, USA).

## Results

### Flow of recruitment and data collection

**Fig. 1:**
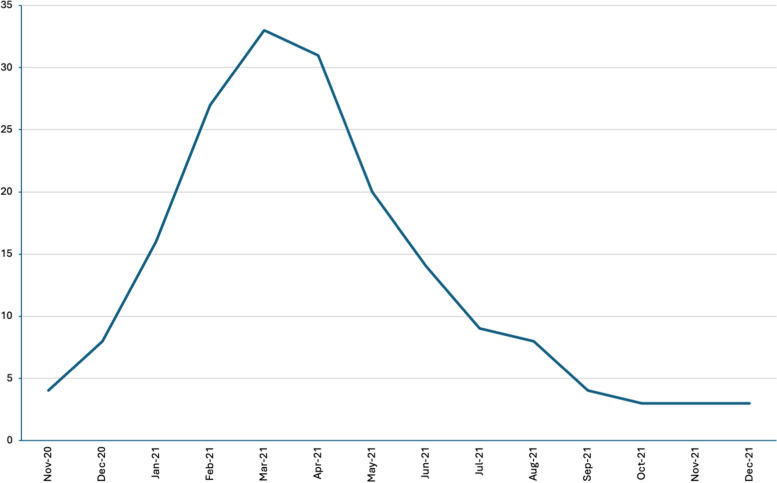
Number of countries per month recruiting general practices between November 2020 and December 2021

Figure 1 shows the flow of recruitment for all 38 countries. Countries collected the data at different points in time. The largest number of countries collected data from January 2021 to May 2021. The number of GP practices who completed at least the first part of the survey was more than 5400 ranging between 13 and 636 across the participating countries.

### Recruitment approaches and participation rate

Based on the responses by each country coordinator, out of the 38 participating countries, 9 (24%) reported that they published an invitation to participate; 19 (50%) had direct contact with all GPs /GP practices; 19 (50%) contacted a sample of GPs /GP practices; and 7 (18%) used another invitation strategy. Table 1 shows the summary of each invitation strategy as reported by each country coordinator. Eleven (30%) of the 38 countries used multiple invitation strategies.

**Table 1:** Invitation strategies used by the country coordinators. Eleven countries used more than one strategy

Invitation strategy	Number of countries reporting this component, n (%)
*Published invitation (N* = *9)*	
Where published?	
As a newsletter (Institutions, Journals)	2 (22.2)
In social media	4 (44.4)
On Medical Association website	2 (22.2)
Multiple venues	1 (11.1)
Who was reached?	
GPs /GPs trainees	9 (100)
Practice managers	6 (66.7)
Other^a^	1 (11.1)
Which region(s) in the country?	
Whole country	7 (77.8)
Specific region(s)	2 (22.2)
*Direct contact with all GP practices (N* = *19)*	
Who was reached?	
GPs /GP trainees	19 (100)
Practice managers	11 (57.9)
Other^b^	1 (5.3)
Οrigin of the contact list	
Government or a governmental organisation	3 (15.8)
National College of GPs (or equivalent)	11 (57.9)
Previous study	3 (15.8)
Known GPs / GPs collaborated with before	9 (47.4)
Other^c^	2 (10.5)
Which region(s) in the country?	
Whole country	17 (89.5)
Specific region(s)	2 (10.5)
How the invitation was sent	
By post	1 (5.3)
By e-mail	18 (94.7)
Participants were reached by phone	1 (5.3)
Other^d^	3 (15.8)
*Contact with a sample of GP practices (N* = *19)*	
Who was reached?	
GPs /GP trainees	18 (94.7)
Practice managers	10 (52.6)
Other	0
Οrigin of the contact list	
Government or a governmental organisation	6 (31.6)
National College of GPs (or equivalent)	6 (31.6)
Previous study	4 (21.1)
Known GPs / GPs collaborated with before	10 (52.6)
Other^e^	3 (15.8)
Sample selection	
Random sample	3 (15.8)
Convenience sample	13 (68.4)
Mixed selection process	3 (15.8)
Sample stratification used?	
No	14 (73.7)
Yes^f^	4 (21.1)
Yes, for part of the sample^g^	1 (5.3)
Which region(s) in the country?	
Whole country	15 (78.9)
Specific region(s)	4 (21.1)
How the invitation was sent	
By e-mail	19 (100)
Participants were reached by phone	3 (15.8)
Other^h^	4 (21.1)
*Other invitation strategy used (N* = *7)*	
Who was reached?	
GPs /GP trainees	6 (85.7)
Practice managers	3 (42.9)
Other	0
Which region(s) in the country?	
Whole country	5 (71.4)
Specific region(s)	2 (28.6)
How the invitation was sent	
By e-mail	6 (85.7)
Participants were reached by phone	2 (28.6)
Other^i^	2 (28.6)

*GP* General practitioner

^a^Germany also included primary care internists

^b^Turkey also included postgraduate physicians

^c^Serbia used a Medical Chamber list; and Turkey used a university list of GP-alumni and GP-trainees

^d^North Macedonia included a viber group of GPs; Bosnia and Herzegovina and Poland did not provide specific information

^e^Czech Republic used a list of the initiative ‘Young Practitioners’; the Netherlands used a sample of practices of the Nivel Healthcare Professionals Registries, and Romania included a list of family doctors from an insurance organization

^f^Kosovo* stratified the sample by gender; Greece and Spain by geographical area; and Austria by gender and geographical area

^g^Belgium stratified part of the sample by geographical area

^h^Spain used Whatsapp; Kosovo* included a printed copy of the questionnaire given in person; Bosnia and Herzegovina and Romania did not provide specific information

^i^Israel printed copies of the survey for participants in conferences; Belgium did not provide specific information

Table 2 shows the estimated number of people reached from countries that reported the same single invitation strategy. The number of people reached from countries that used multiple invitation strategies is shown separately in Table 2.

**Table 2:** Estimates of the number of GP practices invited to participate per invitation strategy

Invitation strategy	Reported number of GP practices invited to participate
*Published invitation only (N* = *2)*	
Participants invited, min – max	1669 – 2580
Participants invited, median (IQR)	2125 (1897, 2352)
*Direct contact of all GP practices only (N* = *12)*	
Participants invited, min – max	130 – 30,000
Participants invited, median (IQR)	950 (560, 1569)
*Contact of a sample of GP practices only (N* = *10)*	
Participants invited, min – max	40 – 873
Participants invited, median (IQR)	331 (104, 575)
*Other invitation strategy used only (N* = *3)*	
Participants invited, min – max	293 – 1270
Participants invited, median (IQR)	746 (520, 1008)
*Countries that used multiple invitation strategies*^*a*^* (N* = *11)*	
Participants invited, min – max	40 – 11,200
Participants invited, median (IQR)	425 (213, 1072)

*IQR* Interquartile range

^a^ Lithuania and Romania used a sample of GP practices combined with another strategy; Luxembourg and Norway used a published invitation combined with direct contact of all GP practices; Bosnia and Herzegovina, North Macedonia, and Serbia used a published invitation combined with a sample of GP practices and with direct contact of all GP practices; Belgium and Israel used a published invitation combined with a sample of GP practices and with another strategy; and Portugal and Ukraine used a sample of GP practices combined with direct contact with all GP practices

Additional file 1 table S4 shows the sampling selection process and the participation rate per country. More than half of the countries [*n* = 21 (55%)] used a convenience sample, 3 (8%) used a mixed sampling process including a random sample; and 3 (8%) countries used only a random sample. Out of the 27 countries that did not invite the total GP practice population, 5 (19%) also used a stratified sample (Additional file 1 table S4). The participation rate ranged from 2 to 94%. Most countries [28, (74%)] reached the target number of participating GP practices (see Additional file 1table S4). The median participation rate among 38 countries was 22% (IQR 10%, 28%).

As shown in Table 3, all countries reported having used at least one strategy to increase the participation rate. Thirty-six (95%) out of the 38 countries sent reminders (usually 1 to 3 reminders per invited GP practice); most of them by e-mail (34 countries; 94%) (Table 3). Twelve (32%) out of the 38 countries used multiple strategies to increase participation (Table 3).

**Table 3:** Strategies used to increase the participation rate

Strategy	Number of countries reported this component, n (%)
Sending out reminders	36 (95)
Number of reminders sent	
Up to 2	15 (42)
Up to 3^a^	11 (31)
Up to 4	5 (14)
At least 4	1 (3)
Up to 5	4 (11)
How the reminders were sent	
By e-mail	34 (94)
Participants were reached by phone	9 (25)
Other ^b^	6 (17)
Giving a financial incentive	1 (3)
Keeping the participant informed about the study results	10 (26)
Offering accreditation points	1 (3)
Other strategy ^c^	6 (16)
Number of strategies used	
One strategy	26 (68)
Multiple strategies	12 (32)

^a^Belgium reported that to some participants, they sent more than 3 reminders

^b^Reminders were sent via Facebook in Denmark; via WhatsApp groups and direct communication in Croatia; while Bosnia and Herzegovina, Kosovo*, Luxembourg, and Norway did not provide specific information

^c^Ireland mentioned the survey at one webinar and a note was placed on the homepage of their website; Poland promoted information about the study on the website of the College of GPs and during its national congress; Bulgaria, Croatia, Germany, and the UK did not provide specific information

Neither the use of multiple invitation strategies nor the use of multiple strategies to increase participation was correlated with the participation rate (see Additional file 1 table S5).

### Representativeness of participating GP practices

Table 4 shows the distribution of the GP practice location in the population and the response group for each country. Across the entire sample, GP practices in towns and suburbs were under-represented, while GP practices in (semi-) rural areas were over-represented (standard chi-square test 443,57; *P*-value < 0.001).

**Table 4:** Urbanisation of the practice location; estimation of the population distribution and distribution in the response group in percentages

Country	Group	Practice location		
		**Big cities**^**b**^	**Town and suburbs**^**b**^	**(Semi-) Rural**^**b**^
Austria	Population	35	25	40
	Response group	**27**	**15**	58
Belgium	Population^a^	25		75
	Response group	52		48
Bosnia and Herzegovina	Population	40	20	40
	Response group	**40**	**30**	**30**
Bulgaria	Population	15	70	15
	Response group	58	15	27
Croatia	Population	30	25	45
	Response group	47	**22**	32
Cyprus	Population	90	9	1
	Response group	53	21	26
Czech Republic	Population	55	25	20
	Response group	**64**	9	**27**
Denmark	Population	35	25	40
	Response group	54	**22**	24
Finland	Population	40	40	20
	Response group	**37**	20	42
France	Population	60	20	20
	Response group	41	**20**	39
Germany	Population	20	50	30
	Response group	**29**	21	50
Greece	Population	14	62	24
	Response group	**16**	12	72
Hungary	Population	39	33	28
	Response group	51	**24**	**25**
Iceland	Population	80	10	10
	Response group	60	27	13
Ireland	Population	35	45	20
	Response group	**39**	20	41
Israel	Population	50	30	20
	Response group	**59**	**22**	**19**
Italy	Population	45	25	30
	Response group	33	**26**	41
Kosovo*	Population	25	15	60
	Response group	50	30	20
Latvia	Population	49	44	8
	Response group	38	26	35
Lithuania	Population	37	58	5
	Response group	**46**	23	31
Malta	Population	20	65	5
	Response group	8	50	42
Moldova	Population	5	12	83
	Response group	22	**17**	62
Netherlands	Population	54	17	29
	Response group	28	**23**	50
Portugal	Population	45	27	20
	Response group	33	**24**	43
Romania	Population	40	27	33
	Response group	75	14	12
Slovenia	Population	40	30	30
	Response group	57	**21**	**30**
Spain	Population	63	36	
	Response group	24	20	55
Sweden	Population	23	50	22
	Response group	**25**	31	44
Switzerland	Population	33	33	33
	Response group	**25**	20	55
Turkey	Population	85	5	10
	Response group	**83**	**2**	**15**
United Kingdom	Population	43	40	17
	Response group	54	29	**17**

^a^Belgium: population data for the whole country (Flanders and Wallonia)

^b^Bold numbers represent ≤ 10%-point or less difference between population and response. Out of 93 cells, 33 are within the 10%-point band. Three countries have all cells within the 10%-point band; four countries have 2 cells within the 10%-point band; 16 countries have 1 cell within the 10%-point band; and 8 countries have no cells within the 10%-point band

Table 5 shows the distribution of the GP practice size in the population and the response group for each country. Across the entire sample, GP practices including more than 10,000 patients, were over-represented, while smaller GP practices were under-represented (standard chi-square test 1549.26; *P*-value < 0.0001).

**Table 5:** Size of the practice based on the number of registered patients or area population; estimation of the population distribution and distribution in the response group in percentages

Country	Group	Practice size				
		** ≤ 2500**^**b**^	**2501–5000**^**b**^	**5000 -10000**^**b**^	**10,001 -50000**^**b**^	** > 50000**^**b**^
Belgium	Population^a^	80	13	5	2	
	Response group	54	28	**15**	**4**	
Bosnia and Herzegovina	Population	90	10			
	Response group	33	30	28	8	3
Bulgaria	Population	80	18	1	1	
	Response group	**72**	**22**	**5**	**1**	
Croatia	Population	98	2			
	Response group	**98**	**2**			
Cyprus	Population	95	1	1	3	
	Response group	**86**	14	**0**	**3**	
Czech Republic	Population	75	20	5		
	Response group	**82**	9	**9**		
Denmark	Population	50	40	9	1	
	Response group	16	54	27	**3**	
Estonia	Population	65	15	10	10	
	Response group	52	**16**	25	**7**	
Finland	Population	2	3	20	55	20
	Response group	81	**9**	0	10	0
France	Population	50	35	10	4	1
	Response group	**51**	**32**	**13**	**3**	**1**
Germany	Population	10	20	60	10	
	Response group	46	32	16	**6**	
Greece	Population	7	5	14	60	14
	Response group	20	**6**	**13**	**54**	**7**
Hungary	Population	97	3			
	Response group	85	14			
Iceland	Population	10	10	20	60	
	Response group	**13**	**10**	**13**	**63**	
Ireland	Population	60	25	10	5	
	Response group	31	**31**	32	**5**	
Israel	Population	80	20			
	Response group	39	33	25	4	
Italy	Population	50	10	40		
	Response group	**42**	27	28		
Kosovo*	Population	55	26	13	4	2
	Response group	**57**	2	**15**	22	**2**
Latvia	Population	95	4	1		
	Response group	**86**	**13**	**1**		
Lithuania	Population	43	21	16	17	2
	Response group	31	**12**	**19**	35	**4**
Malta	Population	90	10			
	Response group	42	**17**	8	25	8
Moldova	Population	85	10	5		
	Response group	17	**15**	29	31	8
Netherlands	Population	28	53	15	4	
	Response group	**29**	**44**	**21**	**6**	
Norway	Population	100				
	Response group	16	45	37	**3**	
Portugal	Population	2	3	30	53	12
	Response group	**1**	**2**	**29**	67	
Romania	Population	3				85
	Response group	18	**1**			72
Slovenia	Population	80	20			
	Response group	96	4			
Spain	Population			46	54	
	Response group	12	**7**	13	67	**1**
Sweden	Population	5	10	80	5	
	Response group	**2**	**11**	38	48	
Serbia	Population	100				
	Response group	27	11	21	26	16
Turkey	Population	60	40			
	Response group	11	21	17	52	

^a^Belgium: population data for the whole country (Flanders and Wallonia)

^b^Bold numbers represent ≤ 10%-point difference between population and response. Out of 155 cells, 58 are within the 10%-point band. Six countries have all cells within the 10%-point band; one country has 4 cells within the 10%-point band; four countries have 3 cells within the 10%-point band; 7 countries have 2 cells within the 10%-point band; 7 countries have 1 cell within the 10%-point band; and 6 have no cells within the 10%-point band

Table 6 shows the distribution of the GP practice type in the population and the response group for each country. Across the entire sample, group practices were over-represented, while solo and duo GP practices were under-represented (standard chi-square test 555.05; *P*-value < 0.001).

**Table 6:** Practice type (solo, duo, or group practice); estimation of the population distribution and distribution in the response group in percentages

Country	Group	Practice type		
		**Solo**^**b**^	**Duo**^**b**^	**Group**^**b**^
Austria	Population	89	8	3
	Response group	65	**18**	16
Belgium	Population^a^	20	20	60
	Response group	36	**20**	46
Bosnia and Herzegovina	Population	25	30	45
	Response group	37	8	**53**
Bulgaria	Population	85	15	
	Response group	**72**	**10**	18
Croatia	Population	95	4	1
	Response group	**93**	**5**	**2**
Cyprus	Population	98	1	1
	Response group	58	29	13
Czech Republic	Population	80	10	10
	Response group	45	36	**18**
Denmark	Population	46	25	29
	Response group	19	**24**	57
Estonia	Population	65	20	15
	Response group	34	**25**	40
Finland	Population	1	1	98
	Response group	13	**9**	71
France	Population	35	20	45
	Response group	**25**	**18**	56
Germany	Population	50	30	20
	Response group	29	**28**	42
Greece	Population			100
	Response group	12	**6**	79
Hungary	Population	23		77
	Response group	87	**10**	3
Iceland	Population			100
	Response group	13	**7**	80
Ireland	Population	20	25	55
	Response group	**15**	**18**	66
Italy	Population	50	10	40
	Response group	39	**13**	**47**
Kosovo*	Population	55	30	13
	Response group	23	3	68
Latvia	Population	99	1	
	Response group	**93**	**5**	**1**
Lithuania	Population	36	22	42
	Response group	4	**23**	73
Malta	Population	80	15	5
	Response group	58		42
Moldova	Population			100
	Response group	22	**8**	69
Netherlands	Population	19	45	36
	Response group	**10**	**36**	54
Norway	Population	10	30	60
	Response group	**4**	4	91
Portugal	Population	2	3	95
	Response group			**100**
Romania	Population	85	10	5
	Response group	**72**	23	5
Slovenia	Population	100		
	Response group	86	**9**	**5**
Spain	Population			100
	Response group	**1**	**2**	**97**
Switzerland	Population	10	25	60
	Response group	27	**22**	49
Turkey	Population	15	40	40
	Response group	**12**	11	74
United Kingdom	Population			100
	Response group	**6**		**94**

^a^Belgium: population data for the whole country (Flanders and Wallonia)

^b^ Bold numbers represent ≤ 10%-point or less difference between population and response. Out of 93 cells, 42 are within the 10%-point band. Five countries have all cells within the 10%-point band; 7 countries have 2 cells within the 10%-point band; 16 countries have 1 cell within the 10%-point band; and 3 countries have no cells within the 10%-point band

Our results did not change when we limited our data to countries that reached the target number of participating GP practices; to countries that invited only a random sample; to countries that invited a mixed sample including a randomly selected sample; and to countries that invited the total number of GP practices. (see Additional file 1 table S6).

### Potential explanations for variation in participation

Across all participating countries, there was no significant correlation between the participation rate and the strength of the PC system [Spearman’s r 0.13, 95% CI (-0.24, 0.46); *P*-value 0.49]. There was no significant correlation between the participation rate and the COVID-19 morbidity [Spearman’s r -0.08, 95% CI (-0.39, 0.24); *P*-value 0.62] and mortality [Spearman’s r 0.05, 95% CI (-0.27, 0.37); *P*-value 0.75] during the first wave. Finally, there was no significant correlation between the participation rate and the COVID-19 morbidity [Spearman’s r 0.19, 95% CI (-0.14, 0.49); *P*-value 0.24] and mortality [Spearman’s r 0.19, 95% CI (-0.02, 0.58); *P*-value 0.06] during the three months preceding the survey.

## Discussion

We start with a brief overview of the main findings for each aim and discuss the findings per aim. Afterwards, we address some more general points of discussion. We will then propose recommendations for future international comparative studies in general practice, based on the experience in the PRICOV-19 study.

### Main findings

Our first aim was to describe the recruitment strategies used. The invitation strategies used in most participating countries in the PRICOV-19 study included a published invitation, such as a newsletter or on social media, contacting all GP practices in the country, or contacting a sample of GP practices. Almost one-third of the countries reported using multiple invitation strategies. All countries used strategies to increase the participation rate, mainly through reminders by e-mail. Recruitment strategies and the combinations used, were diverse, balancing between a consequent strategy according to the study protocol to achieve comparability between all countries and adaption to the local situation. Adaptation to the local situation is crucial and the national coordinators played an important role in deciding on the strategy to use and its implementation. One problem was that – for confidentiality reasons – it was unknown who among the original samples had responded and who had not. Consequently, reminders had to be sent to the whole sample, including the GPs who had already responded. Whether multiple reminders might have been annoying especially for those who had already responded or might have increased the risk of overburdening doctors who were already stretched during to the COVID-19 pandemic remains unclear. However, based on a previous study, multiple reminders did not have a major effect on response patterns [30].

Our second aim was to present the participation rate and the target set in the study protocol. The participation rate varied among countries, with a median of just over 20%. More than two-thirds of the countries reached the target number of participating GP practices, according to the PRICOV-19 protocol. The participation rate was in line with previous surveys among healthcare professionals [31, 32]. In general, using online questionnaires to reach a large sample of the population of interest may be relatively quick and inexpensive but usually results in low response rates [2]. We may think that the digital divide only applies to categories of patients [33]; however, digital abilities might differ among GPs as well and generally be better in some countries than in others [34]. Of course, the study coordinators in the participating countries of the PRICOV-19 study took several steps to maximise participation rates, such as increasing their effort, providing incentives, creating a favourable survey climate, and preparing field workers. Most countries generally managed to overcome difficulties related to the country’s infrastructure and the pandemic burden, and successfully contributed valuable information for the study. Due to the anonymised data collection, the possibility of duplicate responses cannot be excluded. This might have led to an overestimated participation rate and over-coverage error [35]. However, several steps were taken to ensure duplicate responses were not included in the final database.

Our third aim was to assess the relationship between recruitment strategies and the participation rate. Our analysis showed that the participation rate was correlated neither to the use of multiple invitation strategies by the countries nor to the number of additional strategies to increase the response rate. The easy conclusion would be to say that it does not matter what strategy is used; and therefore, to use the cheapest strategy might be considered. However, that would be wrong. The fact that we did not find the expected relationship is probably related to the fact that the country coordinators had to customise their recruitment strategy to the national circumstances. The effect of this customisation can only be assessed in much larger samples of countries or in pooled data sets of comparable recruitment data, to analyse different combinations of strategies.

Our fourth aim relates to the representativeness of the response groups. The distribution of characteristics of the GP practices in the response groups differed from the distribution in the corresponding national populations. Specifically, there was an over-representation of (semi-) rural GP practices, GP practices that included more than 10,000 registered patients, and group GP practices, while there was an under-representation of GP practices located in towns and suburbs, GP practices including fewer registered patients, and solo or duo GP practices. Significant differences between the distribution in the response group and the national population were also found among countries that reached the target number of participating GP practices, invited a random sample, and invited the total population of GPs in the country. Since the information on the characteristics of the GP practices that did not participate was not available for several countries, we attempted to assess representativeness by comparing the response groups of participating GP practices to national population data on the distribution of practice location, size, and type of GP practices. The participating GP practices were not representative of any of these characteristics. A potential explanation might be the differences in implementation of the PRICOV-19 study protocol among countries, which led to over-representation or under-representation of GP practices with certain characteristics. It was probably more practical for practices in semi-rural and rural locations and for practices with more human resources to complete the PRICOV-19 survey than for practices in towns and suburbs, and for small practices with one or two GPs with limited time and support. One might expect better representativeness in countries with larger numbers of respondents; however, this was not the case.

The fifth and final aim was to explain the participation rate. We hypothesized that the strength of the PC system in a country [10], the burden due to the COVID-19 pandemic during the first wave (urgency effect), and during the months before study commencement (urgency or workload effect) might be correlated to the participation rate. Based on our findings, the participation rate was not related to any of these characteristics. However, a potential correlation between higher COVID-19 mortality rates in the 3-month period before the start of the data collection and higher participation rates cannot be excluded. This period differed between countries. Our reasoning was that the COVID-19 mortality in the period just before the start of the survey could indicate the urgency of the situation as well as the workload in the GP practices and that this would influence the willingness to participate. Mortality data are more comprehensive than confirmed cases, as the latter depends on the population’s willingness to undergo a test and the country’s policy regarding testing, but the mortality is also influenced by testing policy [36, 37]. However, the results did not reach statistical significance by the conventional boundary value. We should note here that a high *P*-value does not prove that our groups are equal or that there is no correlation. High *P*-values indicated that our evidence from our sample might not be strong enough to suggest a correlation exists in the population. A correlation between mortality during the three months before the data collection and participation rate might exist, but it is possible that the correlation is too small, that the sample size is too small, or that there is too much variability to detect it. Thus, more participating countries might be necessary to explore potential country-level correlations. It is also likely that the response pattern could be influenced by other unmeasured variables or confounders that increased variability.

### General discussion of important themes

The first issue we want to discuss in more detail concerns using random sampling or a form of convenience sampling. Very few countries implemented a recruitment strategy based on a random sample or used a random sample in addition to other sample selection strategies. Using non-probabilistic sampling methods may yield systematic sampling error by including a fraction of the GP practice population in the country. As in previous studies [10], the country coordinators received uniform instructions for random sampling but also had to consider the feasibility of the suggested procedures in the context of their own country. Moreover, there were no financial resources to support the study implementation in each country. The study was based on the voluntary work of the participating teams. Consequently, the country coordinators had to use their creativity to come as close as possible to the suggested procedures within the restrictions of time, money, and national circumstances and this all happened during the COVID-19 pandemic. To reduce non-response bias [38] in some countries, there has been a stepwise deviation from the original instructions when it turned out that these were not resulting in the expected participation rate.

Related to the choice between random and convenience sampling is the problem of a selective response group that does not reflect the population of all GP practices. Our analysis has shown that the participating GP practices were not representative of specific characteristics of the populations in the participating countries, even for countries that used random sampling. Random sampling is only possible when a sampling frame is available and accessible to researchers. The information about the population distribution was an estimate by the country coordinators in a number of countries due to a lack of official national data on the GP population. Thus, in specific cases there may be a discordance between the expected percentage of certain types of GP practices, e.g., all GP practices should have a population of less than 2500 according to the country coordinator, and the percentage of population categories as reported by participating GP practices from the same country. This observation highlights the importance of the availability to researchers of national registry data that describes important characteristics of the total population of GP practices in each country. That may facilitate researchers to implement probability sampling with appropriate stratification or multi-stage sampling methods, which may yield more representative samples.

We should acknowledge that it is a matter of debate whether it makes much of a difference whether we have a random sample with a very low response rate or a convenience sample. In the end, both are (perhaps equally) biased. Another consideration is that the quality of the answers may be better in a convenience sample; however, as far as we know, research evidence for this is lacking. The common-sense reasoning would be that those who are more involved in a subject, are more motivated to participate, perhaps more knowledgeable about the subject and take more time to fill out the questions meticulously. In a survey like the PRICOV-19 study, we need both an adequate number of respondents and a good quality of data. In addition, we also need the right respondents to minimise selection bias and lack of representativeness. However, despite these problems, the PRICOV-19 study may well suggest answers to specific research questions and contribute to generating new hypotheses [39].

We should bear in mind that these problems are not unique to national surveys among GPs and other PC professionals; however, they are more important in international comparative research [40]. International cross-sectional surveys on general practice may provide valuable data on the organisation and quality of care of GP practices. However, to ensure the generalisability of the findings, we need a well-designed protocol to be uniformly implemented among participating countries. Based on our experience in the PRICOV-19 study, this was difficult to happen. However, it is difficult to generalise from experiences in the PRICOV-19 study to future studies; whether the context and urgency of the COVID-19 pandemic have been a motivator for everybody involved remains unclear.

Despite the suggestions above, a uniform approach to data collection across countries using probabilistic sampling methods might still be difficult. Statistical methods, such as propensity score methods and survey weighting, to achieve unbiased estimates that may be generalisable to the original target population may also have limitations [41]. The differences in the size of the response groups are less of a problem in the analysis of the international survey data, if we apply multilevel analyses (MLA) [42]. Nonetheless, the aims of the PRICOV-19 study were partly descriptive rather than inferential: how do GP practices in different countries organise their work during the pandemic? However, questions that ask for an explanation were also posed. In the explanatory analysis where we look at associations or determinants, the results tend to be less sensitive to lack of representativeness.

### Some practical recommendations for future studies

The recruitment strategy and the realised response – although, relatively high compared to other international surveys of general practice – in the PRICOV-19 study, challenged one of the biggest problems in PC /GP research: How to recruit GPs properly for high-quality research. We have extensively described and analysed the recruitment of GPs in this paper in order to do (even) better in the future. Based on the analysis and the experiences of the partners in PRICOV-19, we propose several recommendations:

- Invest in the motivation of all partners involved; this was done in the PRICOV-19 study through regular consortium meetings, weekly updates, webinars, and meetings (including social events) at conferences during the study [9]. This approach is a possible explanation for the PRICOV-19 study similar participation rates as compared to previous studies [31, 32]. However, future studies may be benefitted from a design that enhances all stakeholders’ engagement from the very beginning of the study according to the relevant published guidelines [43].
- A satisfactory participation rate does not guarantee representativeness of the studied population [44]. Therefore, before the recruitment begins, researchers in all participating countries should ideally agree on which population characteristics the response group needs to be representative for [45]. This will facilitate the stratification and application of survey weights [45].
- Efforts should be made to collect characteristics of the population of GPs and the sample that is approached in each country. Based on the experience in the PRICOV-19 study, including informed estimates from experts when population data are unavailable in a country may sometimes yield in discordances that are difficult to interpret. Thus, all countries should participate in developing core PC characteristics measures that will comply with harmonised, unambiguous definitions. This will help assessing representativeness of the main characteristics between the sampling framework and population data [45].
- Whenever possible, participating countries could include at least a small component of a random sample to assess the presence and potential effects of selection bias [46].
- To our knowledge, this is the first publication addressing the efforts to achieve high response rates to questionnaires through mixed strategies, irrespective of the socio-economic or healthcare or primary care status of the country involved; and thus, it is important that there is replication in a second study in the future. The success of the approach may be likely due to its multifactorialness and the flexibility in its application, since it is probable that some strategies are only used under certain conditions. However, more research would be welcomed to clarify this.
- Our study was focused on PC/GP. However, there is no reason to believe that it would only work with GPs. Other fields in health systems research may also apply these strategies and expand the knowledge of this approach.

## Conclusions

The PRICOV-19 study is the first to provide empirical data from so many countries on how practices responded to the COVID-19 pandemic. It managed to bring 38 countries together to contribute valuable information on the delivery of high-quality care in GP practices, the well-being of the respondents, and possible task changes during the COVID-19 pandemic. Despite all efforts, our work showed that the study might be susceptible to sampling and response bias, and thus, the generalisability of the findings may be compromised. The implementation of an amalgamation of recruitment strategies among countries balancing between a consequent strategy according to the study protocol and adaption to the local situation and the lack of harmonised, unambiguous definitions of major PC characteristics measures are suggested as the main reasons for compromising the PRICOV-19 representativeness. Sample selection and potential bias is an important issue that affects the opportunity for publication in top-rated journals. However, perfect should not be the enemy of good. The PRICOV-19 study comprehensively developed a database including valuable information on GP practice characteristics associated with the quality of provided care and the extended efforts GP practices made to deal with the complexity of the COVID-19 pandemic. In addition, the PRICOV-19 study provided evidence of potential issues that might need attention in the future. To that end, our work was also important in reflecting on what could be done in recruitment and data collection for future large-scale European and other cross-country studies in PC.

### Supplementary Information

Supplementary Material 1.

## Data Availability

The anonymized data is held at Ghent University and is available to participating partners for further analysis upon signing an appropriate usage agreement.

## Abbreviations

CI: Confidence Interval(s)
COVID-19: Coronavirus Disease 2019
GP(s): General Practitioner(s)
IQR: Interquartile Range
MLA: Multilevel Analyses
OECD: Organisation for Economic Co-operation and Development
PC: Primary Care
PR: Participation Rates
REDCap: Research Electronic Data Capture
