# Supplementary material for: Recruitment, data collection, participation rate, and representativeness of the international cross-sectional PRICOV-19 study across 38 countries
Source: BMC Prim Care. 2024 Jun 27;24(Suppl 1):290. doi: 10.1186/s12875-024-02438-w (PMC11212222; doi:10.1186/s12875-024-02438-w)
Supplement: Supplementary file 1 — Supplementary Material 1. [file 12875_2024_2438_MOESM1_ESM.docx]

**Table S1.** Items included in the online questionnaire to describe the invitation strategy and strategies to increase participation rate.

| **Information about invitation strategies answer options** | |
| --- | --- |
| Which strategies did you use to invite participants? | 1. We published the invitation to participate in the  PRICOV-19 study in a local journal, posted it on  Facebook or in a What's app group, on a website,...  2. We directly contacted all GPs/all primary care  practices in the country/in a specific region(s)  in the country and invited them to participate.  3. We directly contacted a sample of GPs/primary care practices in the country/in a specific region(s)  in the country and invited them to participate.  4. Other |
| Follow-up questions: You send published the invitation for this study in a local journal, posted it on Facebook or in a What's app group, on a website ... We would like to learn more about this strategy you used. | |
| - Please describe in detail where you published the  invitation  -Who did you reach with this strategy?  - For which region(s) in your country did you use  this invitation strategy?  -Approximately how many people did you reach using this strategy?  -Are there any other details about this invitation  strategy you used you would like to share with us? | Text box  -General practitioners (GPs) or GP trainees  -Managers of primary care (PC) practices  -Other  Text box  Text box  Text box |
| Follow-up questions: You invited all GPs/PC practices in one or more regions in your country. We would like to learn more about this strategy you used. | |
| -Who did you reach with this strategy?  - To send out the invitations you probably used a list of contact addresses. We would like to know more  about the origin of this list.  - Can you provide us with more details regarding the  origin of the list you used? (e.g. we got the list  from the website of organisation X, we requested the  list from organisation Y by e-mail, ...)  - For which region(s) in your country did you use  this invitation strategy?  -Approximately how many people did you reach using this strategy?  - How did you send the invitation to the study?  - Are there any other details about this invitation  strategy you used you would like to share with us? | -General practitioners (GPs) or GP trainees  -Managers of primary care (PC) practices  -Other  -We used a list that comes from the government or governmental organisation (e.g. downloading it  about the origin of this list. from an official site)  -We used a list that comes from the National  College of GPs (or an equivalent)  -We used a list of contact details originating from  a previous study  - We used a list of contact details of GPs we know  and/or worked with before  - Other  Text box  Text box  Text box  - By post  -By email  -We did not send the invitation but phoned  potential participants  -Other  Text box |
| Follow-up questions: You invited a sample of GPs/PC practices in one or more regions. We would like to learn more about this strategy you used. | |
| -Who did you reach with this strategy?  - What describes best the origin of the list of contact details you used to select the sample?  - Can you provide us with more details regarding the  origin of the list you used? (e.g. we got the list  from the website of organisation X, we requested the  list from organisation Y by e-mail, ...)  - How did you select the sample from this list?  -Did you use a stratification method in selecting  the sample? Please describe in detail: (e.g. the ratio female / male doctors in the country was reflected in the sample)  - For which region(s) in your country did you use  this invitation strategy?  -Approximately how many people did you reach using this strategy?  - How did you send the invitation to the study?  - Are there any other details about this invitation  strategy you used you would like to share with us? | -General practitioners (GPs) or GP trainees  -Managers of primary care (PC) practices  -Other  -We used a list that comes from the government or governmental organisation (e.g. downloading it  about the origin of this list. from an official site)  -We used a list that comes from the National  College of GPs (or an equivalent)  -We used a list of contact details originating from  a previous study  - We used a list of contact details of GPs we know  and/or worked with before  - Other  Text box  Text box  Text box  Text box  Text box  - By post  -By email  -We did not send the invitation but phoned  potential participants  -Other  Text box |
| Follow-up questions: You used 'other' strategies to invite participants in the PRICOV-19 study. We would like to learn more about these strategies | |
| -Who did you reach with this strategy?  - Please describe ‘other’  - For which region(s) in your country did you use  this invitation strategy?  -Approximately how many people did you reach using this strategy?  - How did you send the invitation to the study?  - Are there any other details about this invitation  strategy you used you would like to share with us? | -General practitioners (GPs) or GP trainees  -Managers of primary care (PC) practices  -Other  Text box  Text box  Text box  - By post  -By email  -We did not send the invitation but phoned  potential participants  -Other  Text box |
| Other relevant information |  |
| - Are there any other details about the way you  invited the respondents you would like to share with  us? | Text box |
| **Information about strategies to increase the participation rate** | |
| - Which strategies did you use to replace  non-responders or refusers? | Text box |
| - Which strategies did you use to increase the  participation rate? | -Sending out reminders to the potential participants  Giving a financial incentive  -Keeping the participant informed about the study  results  -Accreditation points  -Other |
| -Approximately how many reminders did you send out? | Text box |
| - How did you send out reminders?  Are there any other details about the strategies  that you used to increase the participation rate that  you would like to share with us? | -By post  -By email  -By phone  -Other  Text box |

**Table S2.** Items included in the online questionnaire on additional information about GP practices characteristics of the total population in each country

| **Queries** | |
| --- | --- |
| Please indicate the country/region in which you have collected data: |  |
| What is the total number of GP practices in your country?  Feel free to clarify your answer | Text box |
| Please estimate the proportion of GP practices per category in relation to the total number of GP practices in your country :  The sum of the percentages that you filled you for the three types (solo, duo, group) should be 100. e.g., in total, there are 5000 GP practices in country X, including 25% solo, 15% duo practices, and 60% group practices)  Feel free to clarify your answer | 1. solo practices (i.e. 1 GP is working in the   practice):……………………..   1. duo practices (i.e. 2 GPs are working in the   practice): ……………………….   1. group practices (i.e. at least 3 GPs are working   in the practice): ………… |
| Please estimate the proportion of GP practices per category in relation to the total number of GP practices in your country: The sum of the percentages that you filled you for the different types of practice size* should be 100. e.g., in total, there are 5000 GP practices in country X, including 20% practices from category 1, 30% from category 2, 10% from category 3, 25% from category 4, and 15% from category 5.  *If there is no registration, please consider the total practice population  Feel free to clarify your answer | 1. up to 2,500 registered patients……………… 2. 2,501 – 5,000 registered participants………… 3. 5,001 – 10,000 registered participants……… 4. 10,000 – 50,000 registered participants…….. 5. More than 50,000 registered participants …… |
| Please estimate the proportion of GP practices per category in relation to the total number of GP practices in your country :  The sum of the percentages that you filled you for the three categories of urbanization should be 100. e.g., in total, there are 5000 GP practices in country X, including 40% practices located in urban areas, 30% practices in small towns, and 30% practices in rural areas.)  Feel free to clarify your answer | 1. in an urban area (big city, suburbs) …………… 2. in small towns…………………………… 3. in rural areas (mixed urban-rural or rural): |
|  |  |

**Table S3.** Strength of primary care system[24-26], urgency effect, and workload effect during first COVID-19 wave[27] and during 3 months before the survey[27] per participating country based on published indicators.

| **Country** | ***Strength of PC*** | **Urgency effect** | | **Workload effect** | |
| --- | --- | --- | --- | --- | --- |
|  |  | **Confirmed COVID-19**  **cases during 1^st^ wave** | **Deaths during 1^st^ wave** | **Confirmed COVID-19 cases 3 mo before survey** | **Deaths 3 mo before survey** |
| Austria | 2.24 | 1781 | 69 | 33,674 | 729 |
| Belgium | 2.23 | 4,698 | 770 | 42,284 | 580 |
| Bosnia and Herzegovina | No information | 685 | 39 | 20,735 | 632 |
| Bulgaria | 2.14 | 310 | 15 | 23,878 | 1,123 |
| Croatia | No information | 544 | 23 | 27,349 | 898 |
| Cyprus | 1.97 | 1,012 | 19 | 27,199 | 204 |
| Czech Rep | 2.16 | 724 | 28 | 46,452 | 734 |
| Denmark | 2.39 | 1,856 | 92 | 22,233 | 261 |
| Estonia | 2.30 | 1,333 | 48 | 7,480 | 41 |
| Finland | 2.31 | 1,122 | 53 | 3,023 | 11 |
| France | 2.17 | 2,686 | 408 | 22,642 | 488 |
| Germany | 2.22 | 2,089 | 94 | 20,039 | 556 |
| Greece | 2.12 | 271 | 15 | 11,568 | 429 |
| Hungary | 2.10 | 355 | 46 | 42,623 | 1,553 |
| Iceland | 1.84 | 4,886 | 27 | 3,015 | 46 |
| Ireland | 2.18 | 4,808 | 305 | 27,001 | 279 |
| Israel | No information | 1,788 | 29 | 23,467 | 205 |
| Italy | 2.34 | 3,709 | 524 | 29,648 | 633 |
| Kosovo* | No information | 513 | 16 | 19,909 | 396 |
| Latvia | 2.17 | 520 | 10 | 20,909 | 320 |
| Lithuania | 2.28 | 565 | 15 | 62,027 | 986 |
| Luxemburg | 1.94 | 6,180 | 164 | 11,537 | 27 |
| Malta | 2.14 | 1,031 | 12 | 22,711 | 393 |
| Moldavia | No information | 1428 | 50 | 20,681 | 407 |
| Netherlands | 2.49 | 2,548 | 329 | 36,082 | 383 |
| North Macedonia | 2.24 | 835 | 47 | 28,624 | 955 |
| Norway | 2.27 | 1504 | 42 | 6,297 | 53 |
| Poland | 2.14 | 477 | 24 | 26,879 | 637 |
| Portugal | 2.41 | 2,811 | 117 | 33,171 | 485 |
| Romania | 2.31 | 859 | 56 | 20,220 | 400 |
| Serbia | No information | 1,519 | 33 | 50,478 | 465 |
| Slovenia | 2.37 | 705 | 50 | 35,065 | 626 |
| Spain | 2.43 | 4,924 | 587 | 33,318 | 480 |
| Sweden | 2.25 | 2,963 | 359 | 39,030 | 605 |
| Switzerland | 2.05 | 3,501 | 203 | 25,708 | 557 |
| Turkey | 2.28 | 1,722 | 48 | 23,901 | 172 |
| Ukraine | No information | 399 | 11 | 14,452 | 346 |
| United Kingdom | 2.51 | 3,322 | 489 | 8,521 | 307 |

PC= primary care; mo, months

**Table S4.** Sample selection process per country and participation rate

| **Country** | **Sample selection** | **Stratification** | **Participating practices** | **Participation rate** | **Target reached** |
| --- | --- | --- | --- | --- | --- |
| Austria | random | Yes (gender and geographical) | 134 | 28% | yes |
| Belgium | random | Partially stratified (geographical) | 451 | 30% | yes |
| Bosnia and Herzegovina | total | Not applicable | 40 | 5% | no |
| Bulgaria | convenience | No | 79 | 94% | yes |
| Croatia | convenience | No information | 140 | 12% | yes |
| Cyprus | total | Not applicable | 11 | 2% | no |
| Czech Republic | random | No | 102 | 22% | yes |
| Denmark | convenience | No information | 37 | 2% | no |
| Estonia | total | Not applicable | 100 | 14% | yes |
| Finland | convenience | No information | 113 | 16% | yes |
| France | total | Not applicable | 603 | 2% | yes |
| Germany | convenience | No information | 249 | 16% | yes |
| Greece | random | Yes (geographical) | 85 | 94% | yes |
| Hungary^1^ | convenience | No information | 220 | 23% | yes |
| Iceland^1^ | convenience | No information | 30 | 24% | no |
| Ireland | total | Not applicable | 183 | 12% | yes |
| Israel | convenience | No | 85 | 22% | yes |
| Italy | convenience | No | 203 | 26% | yes |
| Kosovo* | convenience | Yes (gender) | 60 | 73% | no |
| Latvia | total | Not applicable | 137 | 9% | yes |
| Lithuania | convenience | No | 26 | 23% | no |
| Luxembourg | total | Not applicable | 21 | 5% | no |
| Malta | total | Not applicable | 12 | 7% | no |
| Moldova | convenience | No information | 65 | 24% | yes |
| Netherlands | random | No | 160 | 19% | yes |
| North Macedonia | total | Not applicable | 41 | 93% | no |
| Norway | total | Not applicable | 134 | 11% | yes |
| Poland^1^ | convenience | No information | 196 | 10% | yes |
| Portugal | random | No | 213 | 23% | yes |
| Romania | convenience | No | 97 | 25% | yes |
| Serbia | convenience | No | 101 | 90% | yes |
| Slovenia^1^ | convenience | No information | 180 | 20% | yes |
| Spain | convenience | Yes (geographical) | 294 | 77% | yes |
| Sweden^1^ | convenience | No information | 81 | 7% | yes |
| Switzerland | convenience | No | 85 | 32% | yes |
| Turkey^1^ | convenience | No information | 141 | 28% | yes |
| Ukraine | total | Not applicable | 222 | 2% | yes |
| United Kingdom | convenience | No | 24 | 60% | no |

^1^ These countries clarified that eventually they used a conventional sample instead of inviting the total population as initially was reported in the survey questionnaire

**Table S5.** Use of multiple invitation strategies and use of multiple strategies to increase participation per participation rate quartile

| **Characteristic** | **Participation rate lower than 10.1%**  **[N=9]** | **Participation rate 10.1% - 21.9%**  **[N=10]** | **Participation rate 22% - 28.4%**  **[N=10]** | **Participation rate higher than 10.1%**  **[N=9]** | **P-value^1^** |
| --- | --- | --- | --- | --- | --- |
| Multiple invitation strategies, n (%) | 3 (27.3) | 2 (18.2) | 3 (27.3) | 3 (27.3) | 0.93 |
| Multiple strategies to increase participation rate, n (%) | 2 (16.7) | 3 (25.0) | 4 (33.3) | 3 (25.0) | 0.64 |

^1^ A *P-*value <0.05 was considered as statistically significant

**Table S6.** Examining how well the distribution of practice location, size, and type for all GP practices in participating countries mirrored the distribution of practice location, size, and type in the population GP practices.

|  | **Chi-square^1^** | **P-value** |
| --- | --- | --- |
| *All GP practices in the sample [N=35]^2^* |  |  |
| Distribution of GP practice location | 443.57 | <0.001 |
| Distribution of GP practice size | 1549.26 | <0.0001 |
| Distribution of GP practice type | 555.05 | <0.001 |
| *For countries that reached the target number of participating practices [N=27]* | | |
| Distribution of GP practice location | 445.58 | <0.001 |
| Distribution of GP practice size | 1075.07 | <0.001 |
| Distribution of GP practice type | 557.84 | <0.001 |
| *For countries that invited only a random sample [N=3]* |  |  |
| Distribution of GP practice location | 689.34 | <0.001 |
| Distribution of GP practice size | 323.00 | <0.001 |
| Distribution of GP practice type | 24.44 | <0.001 |
| *For countries that invited a mixed sample including a random sample [N=3]* | | |
| Distribution of GP practice location | 15.40 | <0.001 |
| Distribution of GP practice size | 383.69 | <0.001 |
| Distribution of GP practice type | 45.64 | <0.001 |
| *For countries that invited the total number of practices in the country [N=9]* | | |
| Distribution of GP practice location | 204.56 | <0.001 |
| Distribution of GP practice size | 119.39 | <0.001 |
| Distribution of GP practice type | 42.77 | <0.001 |

^1^ One sample chi-square analysis: standard approach

^2^ Three out of the 38 participating countries did not provide additional information on the population distribution of practices based on location, size, and type
